# Virus-Encoded microRNAs: An Overview and a Look to the Future

**DOI:** 10.1371/journal.ppat.1003018

**Published:** 2012-12-20

**Authors:** Rodney P. Kincaid, Christopher S. Sullivan

**Affiliations:** The University of Texas at Austin, Molecular Genetics & Microbiology, Austin, Texas, United States of America; University of Alberta, Canada

## Abstract

MicroRNAs (miRNAs) are small RNAs that play important roles in the regulation of gene expression. First described as posttranscriptional gene regulators in eukaryotic hosts, virus-encoded miRNAs were later uncovered. It is now apparent that diverse virus families, most with DNA genomes, but at least some with RNA genomes, encode miRNAs. While deciphering the functions of viral miRNAs has lagged behind their discovery, recent functional studies are bringing into focus these roles. Some of the best characterized viral miRNA functions include subtle roles in prolonging the longevity of infected cells, evading the immune response, and regulating the switch to lytic infection. Notably, all of these functions are particularly important during persistent infections. Furthermore, an emerging view of viral miRNAs suggests two distinct groups exist. In the first group, viral miRNAs mimic host miRNAs and take advantage of conserved networks of host miRNA target sites. In the larger second group, viral miRNAs do not share common target sites conserved for host miRNAs, and it remains unclear what fraction of these targeted transcripts are beneficial to the virus. Recent insights from multiple virus families have revealed new ways of interacting with the host miRNA machinery including noncanonical miRNA biogenesis and new mechanisms of posttranscriptional *cis* gene regulation. Exciting challenges await the field, including determining the most relevant miRNA targets and parlaying our current understanding of viral miRNAs into new therapeutic strategies. To accomplish these goals and to better grasp miRNA function, new in vivo models that recapitulate persistent infections associated with viral pathogens are required.

## Introduction

In recent years, non-protein-coding regulatory RNAs have been the subject of increasing interest in both prokaryotic and eukaryotic fields. A new understanding of the mammalian genome is emerging where a majority (50%–85%) of the genome is transcribed with at least some noncoding RNA (ncRNA) transcripts being functionally relevant [1]. Although it is likely that new functions and classes remain to be described, diverse ncRNAs have already been implicated in regulating gene expression at multiple levels, including chromatin modification, transcription, and posttranscriptional mechanisms (reviewed in [2]).

RNA interference (RNAi), the process whereby small ncRNAs (<30 nts) serve to direct gene silencing via specific protein machinery, is evolutionarily conserved throughout most eukaryotes. Discovered in studies of the nematode *C. elegans* [3], with important contributions from the plant and *Drosophila* research communities, RNAi commonly functions to defend hosts against harmful nucleic acids such as endogenous transposons or exogenous viruses (reviewed in [4]–[6]). While the antiviral role of RNAi is well-established in plants, insects, and nematodes, this does not seem to be the case in most (if not all) mammalian cell contexts. When compared to some plants and invertebrates, strong experimental evidence supporting an antiviral role for mammalian RNAi is lacking yet remains the subject of ongoing debate [7]–[9]. Nevertheless, at least some components of the RNAi machinery appear to protect mammalian cells against endogenous transposon activity [10]–[12].

Prokaryotes also possess a nucleic acid-based defense called Clustered Regularly Interspaced Short Palindromic Repeats (CRISPRs). Like RNAi, CRISPRs can be thought of as a nucleic acid-based adaptive immune response providing protection against plasmids, transposons, or phage. Similar to RNAi, some bacterial CRISPR systems use double stranded RNA (dsRNA) and RNAse III enzymes in the process of generating effectors that silence gene expression, typically through cleavage of targeted DNA [13]. Functional CRISPR machinery has been lost or gained numerous times in bacterial lineages. Similarly, RNAi has been lost in some eukaryotic lineages including the important model organism *Saccharomyces cerevisiae*, and some loss-of-functions in either RNAi or CRISPRs have been associated with the gain of beneficial foreign genetic elements [14]–[16]. It has been proposed that some bacteria evolved to adapt CRISPR machinery to regulate self protein-coding gene expression [17]. Similarly, at least once and possibly multiple times, eukaryotic lineages have evolved to use components of the RNAi machinery to regulate self protein-coding gene expression via a class of small RNAs called microRNAs (miRNAs) [18].

**Figure 1:**
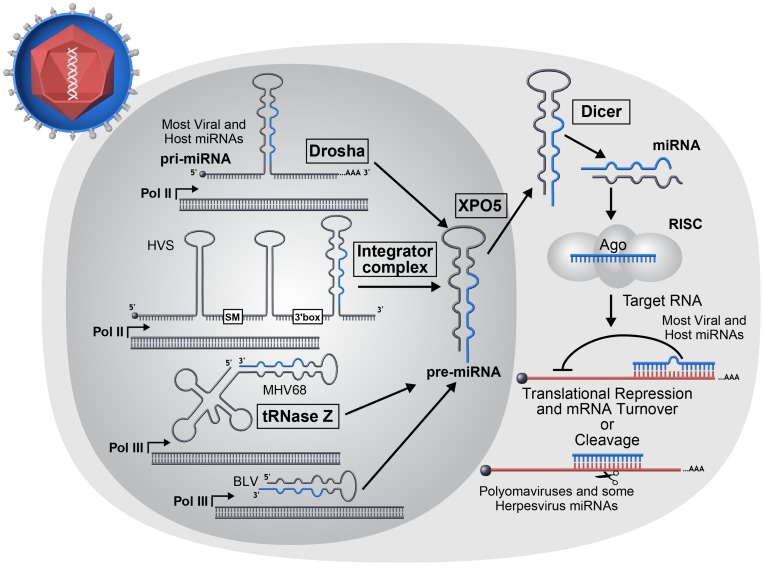
miRNA biogenesis overview. The majority of host and viral miRNAs begin as longer RNAs (pri-miRNAs) transcribed by host RNA Pol II that are recognized and processed by the host Microprocessor complex to produce a short stem-loop RNA (pre-miRNA). However, a minority of viruses utilize noncanonical mechanisms in the biogenesis of pre-miRNA molecules. Herpesvirus saimiri (HVS) encodes Sm class U RNAs (HSURs) that are transcribed by RNA Pol II and subsequently processed by the host Integrator complex to generate pre-miRNAs [115]. The miRNAs encoded by mouse gammaherpesvirus 68 (MHV68) and BLV are both transcribed by host RNA Pol III [35], [39], [116], [117]. The MHV68 miRNAs are processed from larger tRNA-like RNAs by host tRNase Z and possibly additional factors to generate pre-miRNAs. Pre-miRNAs are exported to the cytoplasm where they are cleaved into short ∼22 nt duplex RNAs by the host enzyme Dicer. One strand of the duplex may be incorporated into an Argonaute protein containing effector complex known as RISC. The incorporated miRNA directs RISC to target RNAs. Most commonly for animal host and virus-encoded miRNAs, imperfectly complementary base pairing occurs between miRNA and mRNA target resulting in translation inhibition and mRNA turnover. However rarely, perfect base pairing can occur resulting in siRNA-like RNA cleavage and has been reported for polyomaviruses and some herpesviruses.

miRNAs are small, approximately 22 nt RNAs that typically silence gene expression by directing repressive protein complexes to the 3′ untranslated region (UTR) of target messenger RNA (mRNA) transcripts. The first miRNAs were discovered in *C. elegans* via forward genetic screens designed to identify genes involved in larval stage development [19]. Years later, three seminal papers demonstrated that miRNAs represent a large family of genes, some of which are evolutionarily conserved among insects, nematodes, and humans [20]–[22]. Since their discovery, interest in miRNAs has grown at an exponential rate. Numerous processes, many of clinical importance, are regulated by miRNAs. Of particular relevance to host–pathogen interactions, miRNAs play a role in regulating the innate immune response, adaptive immune cell differentiation, metabolism, apoptosis, cell proliferation, cancer, and maintenance of homeostasis during stress. Canonical miRNAs derive from longer precursor primary transcripts (pri-miRNAs) that are typically transcribed by RNA polymerase II (pol II) (Figure 1). Pri-miRNAs contain at least one, but often several, precursor(s) of imperfectly complementary stem-loop hairpin structures. In mammals, the precursor miRNAs (pre-miRNAs) are liberated from the larger pri-miRNA via the RNAseIII-like endonuclease Drosha ([23] and references therein). Drosha, along with its binding partner DGCR8 (Pasha in *Drosophila*), comprise the Microprocessor complex that binds to the pri-miRNA, where multiple structural cues position cleavage towards the base of the hairpin stem. The newly liberated ∼60 nt hairpin pre-miRNA is then exported from the nucleus to the cytoplasm via the RAN-GTPase Exportin 5. Once in the cytosol, the pre-miRNAs are cleaved by the RNAse III-like endonuclease Dicer. Dicer-mediated cleavage produces a transient ∼22 nt duplex RNA, of which one strand (the miRNA or “guide” strand) is stably incorporated into the RNA-induced silencing complex (RISC). The other strand, called the “star” (*) or “passenger” strand, is less likely to associate with RISC and consequently is typically found at several-fold lower steady state levels. RISC is a multiprotein complex of which a key component is an Argonaute (Ago) protein. Ago-loaded miRNAs (miRISC) typically bind to target transcripts and repress gene expression. However, notable exceptions including translational activation under stress conditions and modulation of hepatitis C virus (HCV) replication have been reported [24], [25].

Mounting evidence suggests that miRISC functions by inducing an initial blockade to translation followed by enhanced turnover of repressed target transcripts [26], [27]. Because there are potentially hundreds of miRNAs of biological relevance in any given cell type, each with the potential to regulate many (some studies suggest >100) target transcripts, a model has emerged portraying a complex web of posttranscriptional regulation comprised of numerous interconnected miRNAs and targets (reviewed in [28]). In this model, miRISC complexes loaded with different miRNAs form the “nodes” of the web, with each miRNA regulating numerous transcripts. Conversely, each transcript is capable of being regulated in an additive fashion by different miRISC complexes. Thus, even though a typical miRNA may impart only a relatively modest effect on any single target, the sum total of transcript regulation conveyed by a particular miRNA can combine for significant phenotypic consequences. On the other hand, data exist that for some miRNAs, only a minority of miRISC-mRNA target interactions are of biological importance (reviewed in [29]). Additionally, gene knockout studies in animals demonstrate that numerous miRNAs are not essential for viability [30], [31]. This suggests that some miRNAs serve a primary role as subtle regulators to “fine tune” or “balance” levels of gene expression. Consequently, some miRNAs are only essential during stress, serving as key mediators of homeostasis. Therefore, host miRNAs display a spectrum of gene regulatory activities with phenotypic consequences ranging from subtle to profound, and it may be expected that virus-encoded miRNAs will behave the same.

## Virus-Encoded miRNAs

### Which Types of Viruses Encode miRNAs?

**Figure 2:**
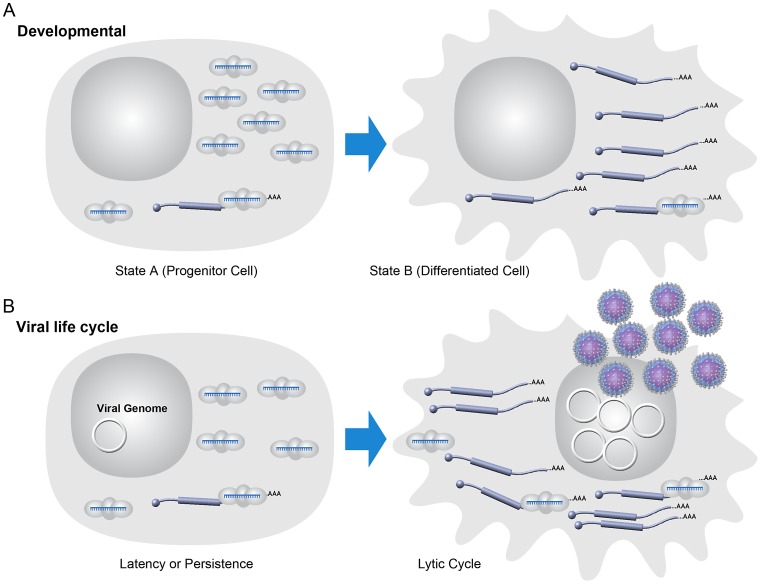
Model: viral latency as a simple developmental process. (A) Some host developmental pathways may be modeled in a two-state fashion. Host miRNAs may help enforce or sharpen transitions from one developmental state to another with miRNA and mRNA target levels inverted in those states [94]. (B) Model for some miRNAs during viral latency. During viral latency, lytic mRNAs are not expressed or expressed at typically undetectable levels. Viral miRNAs are expressed at relatively high levels during latency and may suppress “leaky” lytic transcripts. Optimizing the switch from latent to lytic infection is likely a function of importance in maintaining the homeostasis of latent infection. During lytic replication, large changes in the transcriptional activity of lytic genes “overrun” the imposed inhibition by miRNAs. Note that the viral genome is represented as a circular episome even though for some viruses lytic replication can result in multiple copies of the linearized genome.

RNAi likely arose as a primary defense against harmful genetic elements such as viruses, yet in an interesting evolutionary twist, divergent viruses co-opted miRNA expression for pro-viral purposes. DNA viruses account for the majority of known virus-encoded miRNAs with the herpesvirus family encoding most known viral miRNAs. Herpesviruses dominate both in terms of absolute number of known virus-encoded miRNAs and in the average number of miRNAs encoded per virus (typically >10/genome). Herpesviruses comprise an extended family of large genome DNA viruses whose defining trait is the ability to undergo long-term, and often life-long, latent infections. Latency is a specialized type of persistent infection where only a few viral gene products are expressed allowing for efficient evasion of the immune response. Latency is fully reversible, and with appropriate cues, the virus initiates the lytic mode of infection comprising full viral gene expression and culminating in the production of infectious virus and lysis of the host cell [32]. In this regard, the dual infectious modes of herpesviruses (latent versus lytic) can be thought of as a very simple two-stage model—similar to cell type differentiation that occurs in eukaryotic organisms during development. Both processes integrate extracellular events and subsequent signal transduction. Both ultimately depend on sometimes subtle differences in gene expression that are regulated by transcriptional and/or posttranscriptional mechanisms (Figure 2).

Several aspects of the herpesvirus life cycle are instructive for understanding the other virus families that encode miRNAs. miRNAs are likely invisible to the adaptive immune response—a valuable trait for viruses that undergo persistent infection [7]. Given the often subtle nature of miRNA-mediated regulation, it is likely that most virus-encoded miRNAs may have a diminished role during lytic infection where robust changes in host and viral gene expression dominate even though viral miRNAs are typically detectable at these times. For the most part, natural viruses that encode miRNAs have a DNA component to their replication cycle, replicate in the nucleus where they have full access to the initiating host miRNA biogenesis machinery, and undergo long-term persistent infections. These include viruses with DNA genomes (The Herpesvirus, Polyomavirus, Ascovirus, Baculovirus, Iridovirus, and Adenovirus families) and at least one member of the retrovirus family, bovine leukemia virus (BLV) (Table 1).

**Table 1:** Known viral miRNAs and proposed functions highlighted in this review.

Virus Family or Subfamily	Virus Species	Pre-miR Hairpins	Mature miRs	Proposed Functions Highlighted in this Review
Alpha-herpesvirinae	Herpes Simplex Virus 1	16	25	
	Herpes Simplex Virus 2	18	24	
	Herpes B virus	>3^a^	>3^a^	
	Herpesvirus of turkeys	17	28	
	Infectious laryngotracheitis virus	>7^a^	>10^a^	
	Bovine herpesvirus 1	10	12	
	Marek's disease virus type 1	14	26	Prolonging longevity of infected cells [57], host miR-155 mimic [28], [67]
	Marek's disease virus type 2	18	36	Host miR-29 mimic
	Pseudorabies virus	13	13	
Beta-herpesvirinae	Human cytomegalovirus	11	17	Prolonging longevity of infected cells [61]
	Mouse cytomegalovirus	18	28	Evasion of the immune response [84]
	Human herpesvirus 6B	4	8	
Gamma-herpesvirinae	Epstein–Barr virus	25	44	Prolonging longevity of infected cells [54]–[56], [62], host miR-29 mimic
	Rhesus lymphocryptovirus	36	50	Host miR-29 mimic
	Kaposi's sarcoma-associated herpesvirus	12	25	Prolonging longevity of infected cells [58]–[60], regulating host and viral genes to limit the lytic cycle [87]–[91], host miR-155 mimic [68], [72]–[74]
	Rhesus monkey rhadinovirus	15	25	
	Herpesvirus saimiri strain A11	3	6	
	Mouse gamma herpesvirus 68	15	28	
Polyomaviridae	Simian virus 40	1	2	Autoregulation of viral early genes [79]
	JC polyomavirus	1	2	Autoregulation of viral early genes [96], evasion of the immune response [83]
	BK polyomavirus	1	2	Autoregulation of viral early genes [96], evasion of the immune response [83]
	Mouse polyomavirus	1	2	Autoregulation of viral early genes [80]
	Merkel cell polyomavirus	1	2	Autoregulation of viral early genes [97]
	SA12	1	2	Autoregulation of viral early genes [99]
Retroviridae	Bovine leukemia virus	5	8	Host miR-29 mimic [39]
Iridoviridae	Singapore Grouper Iridovirus	14	15	
Ascoviridae	Heliothis virescens ascovirus	1	1	Targets viral polymerase transcript [101]
Baculoviridae	Bombyx mori nucleopolyhedrosis virus	4	4	
Adenoviridae	Human adenoviruses types 2 and 5 (others likely)	2^b^	3	
Unclassified	Bandicoot papillomatosis carcinomatosis virus type 1	1	1	Autoregulation of viral early genes [98]
	Bandicoot papillomatosis carcinomatosis virus type 2	1	1	Autoregulation of viral early genes [98]
	Heliothis zea nudivirus-1	2	2	Promotes latency-like state by inhibiting viral gene expression [86]

a Currently annotated miRNAs in miRBase. Recent reports indicate these numbers to be higher [118], [119].

b Note that the Adenoviral miRNAs are derived from inefficient processing of an atypical precursor structure known as the Virus-associated RNAs (vaRNAs).

Naturally occurring positive or negative sense RNA or dsRNA genome viruses that express miRNAs are not widely accepted. In fact, until recently, it had been speculated that viruses with RNA genomes would not encode miRNAs due to negative effects on fitness that would be incurred with *cis* cleavage of the genome, antigenome, or mRNAs mediated by the miRNA processing machinery [33], [34]. Retroviruses package an RNA genome into the capsid but also contain a DNA stage in their infectious cycle where the reverse-transcribed provirus genome integrates into host DNA. It has been reported that HIV may encode miRNAs, but this is not widely accepted due to low abundance, lack of evolutionary conservation amongst strains, unknown biological relevance, and the discordance of results amongst different labs [35]–[38]. BLV, however, clearly encodes numerous miRNAs [39]. Interestingly, BLV avoids Drosha-mediated cleavage of its genome and mRNAs, which overlap the miRNA cluster portion of the genome. This occurs because, unlike most known miRNAs, BLV miRNAs are encoded as shorter RNA polymerase III (pol III) transcribed hairpins that can directly serve as Dicer substrates. As a result, BLV transcripts are not cleaved by Drosha, and only subgenomic small RNAs are processed into miRNAs. Thus, at least one retrovirus encodes miRNAs. Combined with recent reports of laboratory engineered RNA viruses that successfully express miRNA-like RNAs [40]–[43], it seems likely that additional RNA virus-encoded miRNAs await discovery.

As mentioned above, the viruses most likely to encode miRNAs will have nuclear and DNA components to their lifecycle and have the ability to establish persistent infections. However, it's clear that not all viruses that meet these criteria encode miRNAs. As least one type of human papillomavirus (HPV, small dsDNA genome viruses some of which are associated with human tumorigenesis) does not encode miRNAs [44]. We note that these findings do not exclude the possibility that other PVs may encode miRNAs. In fact, one study claims that HPV-18 encodes a miRNA but lacks solid proof demonstrating the involvement of the miRNA biogenesis or effector machinery [45]. The preponderance of evidence suggests that at least some PVs, and perhaps many if not all others, do not encode miRNAs. Similarly, although most herpesviruses that have been examined in-depth encode miRNAs, it appears that Varicella Zoster Virus (VZV), the etiologic agent of chicken pox and shingles, does not [46]. This finding is particularly interesting given that other human neurotropic herpesviruses (HSV1 & 2) and, furthermore, other animal Varicelloviruses including Bovine Herpesvirus 1 and Suid Herpesvirus 1 do encode miRNAs [47], [48]. This raises the question as to what is different between the VZV and other herpesviruses lifecycles that determines miRNA utilization. As more small RNA sequencing studies are performed, understanding which viruses do and do not encode miRNAs will be informative to the overarching goal of understanding virus miRNA function.

### Virus-Encoded miRNA Functions

**Figure 3:**
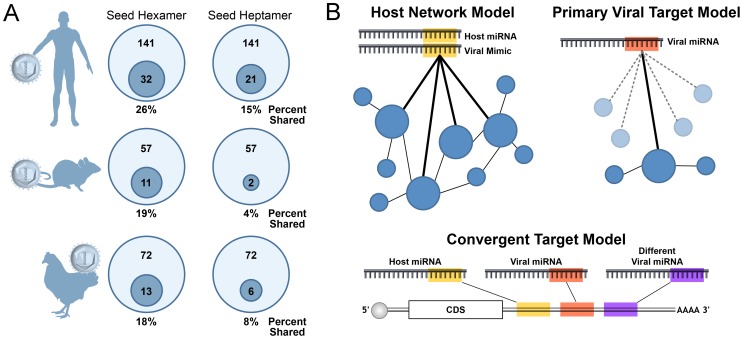
A minority of viral miRNAs mimic host miRNAs through identical seed sequences. (A) Some viral miRNAs share seed identity with host miRNAs, called “analogs,” while the majority of viral miRNAs do not. For each system (human, mouse, chicken), the miRBase version 18 annotated mature viral miRNA sequences were compared with respective host miRNAs for identity in nucleotides 2–7 (hexamer) or 2–8 (heptamer). Inner circles represent the number of viral miRNAs with a host seed match out of the total viral miRNAs. Percentage to the nearest whole number is presented below each diagram. (B) Models of viral miRNA function. In the host network model on the left, some viral miRNAs function as analogs of host miRNAs through seed sequence similarity, thereby targeting transcripts through the same docking sites as the mimicked host miRNAs. These docking sites for the host miRNA may represent a conserved network and allow the viral miRNA access to numerous targets working together to effect the same function. In contrast, the primary target model on the right suggests some viral miRNAs evolve to target only one or a few transcripts through novel sites not conserved for host miRNA functions. In this model, the virus may tolerate numerous neutral or disadvantageous “bystander” interactions as long as the sum total of regulation provided by the nonanalog viral miRNA is advantageous to the viral lifecycle. Additionally, host and viral miRNAs may target the same transcript through different docking sites as proposed in the convergent target model (bottom of figure).

Virus-encoded miRNAs can be grouped into two classes: those that are analogs of host miRNAs and those that are viral specific. Similar to some virus-encoded regulatory proteins, a subset of viral miRNAs have evolved to mimic host effectors. Viral miRNAs that mimic host effectors are referred to as “analogs.” The 5′ end of a miRNA (∼nucleotides 2–8), called the “seed” region, plays an especially important role in directing RISC to mRNA targets. It is estimated that ∼60% of regulation by a particular miRNA is due to binding with perfect seed complementary to the target transcript [49]. A fraction of virus-encoded miRNAs share seeds with host miRNAs and at least three viruses: Kaposi's Sarcoma-associated Herpesvirus (KSHV), Marek's Disease Virus 1 (MDV1), and BLV have been shown to negatively regulate transcripts via the same target docking sites as their counterpart host miRNAs [28], [39]. Mimicking a host miRNA allows a viral miRNA to potentially regulate hundreds of transcripts that have evolved target sites for a particular host miRNA. Presumably such regulatory networks evolve to effect specific functions, for example inhibiting apoptosis. Our estimates suggest ∼26% of currently annotated human virus-encoded miRNAs could mimic host miRNAs by possessing identical seed sequences (Figure 3A). However, this likely represents a gross overestimate because it includes star strands for both host and viral miRNAs. Furthermore, this estimate is based on hexameric seeds, whereas heptameric seeds are better predictors of shared targets [50]. Performing the same analysis using heptameric seeds further reduces the overlap of host and viral miRNA seeds to ∼15%. Similar results are obtained in other systems including viruses with rodent and avian hosts (Figure 3A). Additionally, based on low abundance, untested biogenesis, and unknown functional relevance, it's unclear whether all of the currently annotated viral or host miRNAs are bona fide miRNAs, underscoring that some seed matches between host and viral miRNAs arise by chance [28]. Therefore, it seems likely that only a minority of virus-encoded miRNAs truly mimic host miRNAs.

Although more than 250 virus-encoded miRNAs are known [51], an in-depth functional understanding is lacking for most. Part of this stems from the fact that these miRNAs were only relatively recently discovered. Additionally, there is a lack of easily accessible animal models for some viruses that encode miRNAs, and most viral miRNAs encoded by human viruses are not conserved in nonprimate animal virus models. Based on what is currently known about virus-encoded miRNAs (reviewed in-depth in [28]), we propose the hypothesis that, despite often being detectable during lytic infection, most will function to foster persistent/latent infections. Accordingly, most functions ascribed to virus-encoded miRNAs can be grouped in the following categories: (1) prolonging longevity of infected cells, (2) evading the immune response, and (3) regulating host or viral genes to limit the lytic cycle.

### Prolonging Longevity of Infected Cells

Preventing cell death seems like an obvious advantage to viruses that take up persistent or latent infections in long-lived cells. Host miRNAs play a major role in maintaining cellular homeostasis, and not surprisingly, numerous host miRNAs are implicated in the regulation of cell death (reviewed in [52]). Several different viruses including KSHV, Epstein Barr virus (EBV), and MDV1 encode miRNAs that can play a subtle role in preventing apoptosis by targeting pro-apoptotic host genes. EBV is a gamma herpesvirus associated with cellular hyperproliferative disorders such as infectious mononucleosis as well as B cell and solid cell tumors (reviewed in [53]). The EBV-encoded miRNA miR-BART5 targets the transcript of the pro-apoptotic host gene PUMA [54], and members of the EBV BART miRNA cluster also target transcripts of the pro-apoptotic gene Bim [55]. Furthermore, the EBV BHRF1 miRNAs have been implicated in preventing apoptosis during infection of cultured primary B cells [56]. MDV1-encoded miRNA miR-M3 targets the transcript of host gene Smad2 and has been shown to reduce drug-induced apoptosis in cell culture [57]. Interestingly, for some viruses such as KSHV, different viral miRNAs can target independent host transcripts preventing early initiating apoptotic events such as the cytokine signaling receptor TWEAKR as well as late apoptotic effectors such as caspase 3 [58], [59]. It is also noteworthy that at least three different human herpesviruses (human cytomegalovirus [HCMV], EBV, and KSHV) have been shown to encode miRNAs that target host pro-apoptotic gene BclAF1. These viral miRNAs utilize different miRNA target sites, which may imply that BclAF1 is an important effector in the life cycle of diverse herpesviruses and viral miRNAs may converge on similar targets without reliance on conserved target sites [60]–[62]. Alternatively, it remains possible that since BclAF1 has an atypically long 3′ UTR (>4 Kb), it may be a member of a class of hypothetical transcripts that due to abundance or composition of UTRs are hyper-prone to miRNA-mediated regulation. Although the in vivo relevance remains to be determined, some viral miRNAs likely serve to evade cell death.

Several viruses known to encode miRNAs, including herpesviruses KSHV, EBV, MDV1, and the polyomavirus Merkel Cell Carcinoma Polyomavirus (MCPyV), are associated with tumorigenesis. Inducing tumors is likely not a primary advantage for these viruses but rather an accidental off consequence of the need to alter the cell cycle, prevent cell death, and avoid the immune response [63]. EBV, the etiologic agent of various human tumors, encodes miRNAs that have been implicated in cell culture models of transformation [64]–[66]. In addition, two important in vivo studies have demonstrated a role for MDV1 and KSHV miRNAs in tumorigenesis [67], [68]. Both MDV1, an alpha herpesvirus of chickens, and KSHV, a lymphotropic gamma herpesvirus of humans, are associated with tumors. MDV1 causes T-cell lymphomas and KSHV is associated with a subset of primary effusion lymphomas and Kaposi's Sarcoma (KS). Amazingly, both viruses encode miRNAs that function as analogs of the host miRNA miR-155. Misexpression of miR-155 alters lymphopoeisis and plays a role in tumorigenesis (reviewed in [69]–[71]). Infection of chickens with a mutant version of MDV1 that does not express the viral analog of miR-155 results in loss of oncogenecity in most subjects [67]. Several studies have combined to show that the KSHV analog of miR-155 (miR-K12-11) shares overlapping targets with the host miRNA [72]–[74]. Recently, in an orthotopic humanized mouse model, exogenous expression of KSHV mir-K12-11 was shown to be sufficient to drive hyperproliferation of B cells [68].

We have shown that one of the BLV miRNAs functions as an analog of the host miRNA miR-29. miR-29 has been shown to function as either an oncogene or a tumor suppressor depending on the context [75]. miR-29 is overexpressed in human chronic lymphocytic leukemias (CLLs), which bear a striking phenotypic resemblance to BLV-associated tumors in cattle [76]. When miR-29 is experimentally overexpressed in B cells, mice develop B cell tumors that strongly resemble CLL [77]. Exactly how BLV causes tumors has remained enigmatic since most BLV tumor cells do not express abundant viral pol II transcripts or proteins. The identification of a BLV pol III–derived potential oncomiR supports a model for miRNAs in BLV-mediated tumorigenesis, but this speculation awaits confirmation in vivo. Also worth noting is that at least three other lymphotropic viruses, EBV (miR-BART1-3p), RLCV (Rhesus Lymphocryptovirus, miR-rL1-6-3p), and MDV2 (Marek's Disease Virus 2, miR-M21), also encode miRNAs with miR-29 seeds. Thus, a picture is emerging whereby miRNAs encoded by tumor viruses can contribute to increased cell survival and tumorigenesis.

### Evading the Immune Response

Akin to nonstructural viral proteins that often function to evade the immune response, it seems likely that some viral miRNAs perform a similar role. Theoretically, miRNAs can contribute to immune evasion indirectly by lowering viral protein levels and consequent antigenicity, or directly by suppressing components of the host immune response [78]. Simian Vacuolating Virus 40 (SV40), a prototypic polyomavirus with a circular genome possessing opposing transcriptional units for the early and late genes, encodes a miRNA that is perfectly complementary to the early viral transcripts. The SV40 miRNA directs cleavage of the early viral transcripts and results in reduced early viral gene expression at late times of lytic infection [79]. When SV40-infected cells are co-cultured with cytotoxic T cells (CTLs), more CTL-mediated lysis is observed in cells infected with a miRNA mutant virus. This suggests a possible role for the SV40 miRNA in evading the adaptive immune response in vivo. Murine polyomavirus (muPyV) also encodes a miRNA that negatively regulates early gene expression in a manner similar to SV40 [80]. However, infection of mice with a muPyV miRNA mutant virus does not support a robust role for the miRNA in evading the adaptive immune response as little difference in CTL response is observed [80]. Although SV40 and muPyV are different viruses, they share numerous similarities in infectious cycles and autoregulatory activity of their respective miRNAs. These results suggest that the underlying purpose of Polyomaviridae miRNA-mediated autoregulation remains to be determined and that caution is warranted when interpreting the in vivo relevance of cell culture experiments. miRNAs from several different human herpesviruses and the star strand derivative of the human polyomavirus JC (JCV) have been implicated in co-culture experiments in evading the Natural Killer (NK) cell innate immune response [81]–[83]. While these findings await confirmation in vivo, they do suggest a possible shared function in evading NK cells via miRNAs encoded by very different kinds of viruses. To date, the only in vivo evidence providing a link between miRNAs and immune evasion are from studies conducted on murine cytomegalovirus (MCMV). Deletion of two MCMV miRNAs results in reduced titers in the salivary glands of specific genetic backgrounds of mice [84]. The phenotype, which suggests that these miRNAs function to promote persistent infection, was reverted in mice that were defective in both the adaptive and innate arms of the immune response through the depletion of NK cells and CD4^+^ T-cells. Taken together, these observations support the hypothesis that some virus-encoded miRNAs serve a subtle role in evading the immune response.

### Regulating Host or Viral Genes to Limit the Lytic Cycle

The restricted gene expression of latency and some other forms of persistent infection represents a successful immune evasion strategy. In addition to encoding direct modulators of the immune response, latently infected cells evade the immune response by expressing a limited number of proteins, providing for reduced antigenicity. Several different herpesviruses encode miRNAs that have been implicated in maintaining latent infection and altering the balance between latent/lytic infection [85]. HSV1, KSHV, and HCMV all encode miRNAs that have been reported to subtly regulate either viral and/or host genes that could promote latent/persistent infection (reviewed in-depth [28], discussed briefly below). A recent study demonstrates that miRNA-mediated promotion of latency is not restricted to herpesviruses as Heliothis zea nudivirus-1 (HzNV-1), a large DNA genome insect virus, encodes miRNAs that promote a latency-like state by directly inhibiting viral gene expression [86].

Studies of the KSHV latency system provide some of the most well-documented examples of viral miRNAs regulating the latent/lytic switch (reviewed in-depth in [28]). It should be noted, however, that the effects of individual KSHV miRNAs in this process are invariably subtle. The KSHV encoded miRNAs miR-K12-9-5p and miR-K12-7-5p have been shown to directly regulate the transcript of the master lytic switch protein (RTA) [87], [88]. Several KSHV-encoded miRNAs also target host transcripts that result in enhanced latency [89]–[91]. For example, the KSHV-encoded miRNA, miR-K12-1-5p, directly targets the transcript of host gene IκBα, which modulates the NF-κB pathway and reduces lytic activation [89]. Additionally, miR-K12-3-5p directly targets the transcript of host transcription factor NFIB, which has been shown to be an activator of the RTA promoter [91]. Importantly, a KSHV deletion mutant that removes most of the viral miRNAs or knockdown of KSHV miRNA function results in increased lytic activity [89], [91]. As mentioned above, although not as well studied as KSHV, it is likely that virus-encoded miRNAs from some other herpes viruses also regulate entry into or the degree of lytic infection.

Large-scale efforts to identify transcripts directly targeted by herpesvirus miRNAs have implied that at least some putative viral lytic replication-inducing targets are not detectable in association with RISC [62], [92], [93]. This may suggest that these putative targets are not biologically relevant. On the other hand, the lack of sensitivity of these target identification methods likely would have missed very low abundance “leaky” transcripts. As low abundance lytic-promoting transcripts may be sufficient to initiate feed-forward lytic-inducing loops, a role for viral miRNAs in promoting latency cannot be ruled out from these negative target profiling studies. Indeed, host miRNAs generally display an inverse cell type expression profile with their targets, suggesting that a major role of miRNAs is to enforce homeostasis when inappropriate low-level “leaky” transcripts are expressed [94], [95]. By analogy, enforcing latency against low-level gene expression noise could very well also apply to virus-encoded miRNAs and the simple developmental state of latency (Figure 2).

What about those viruses without a well-defined latency? Polyomaviruses generally take up life-long persistent infections in their hosts but the mechanisms that allow for this are not well understood. We have shown that several polyoma and polyoma-like viral miRNAs regulate or have the capacity to regulate early gene expression during late times of lytic infection [79], [96]–[99]. Although regulation during lytic infection could be the main function of these miRNAs, it is also possible that, similar to the herpesviruses and HzNV-1, polyomaviral miRNAs may play a role in tilting the balance between persistent and lytic infection. Similarly, HCMV miRNAs have not been studied in a latent context, but it could be predicted based on known viral targets of HCMV [100] and the limited understanding of MCMV miRNA function in vivo [84] that some HCMV miRNAs will play a role in maintaining or establishing latency/persistence. The DNA insect virus Heliothis virescens ascovirus (HvAV) encodes a miRNA that targets its own viral polymerase [101]. Although HvAV infection results in death in some insect hosts, it has recently been shown that other insect hosts may undergo an attenuated infection and thus serve as possible reservoirs for persistent infection [102]. Therefore, it is possible that the HvAV miRNA could promote persistent infection in some contexts. Testing a role for the polyomaviral, HCMV, and ascoviral miRNAs in promoting persistence will likely require developing sensitive in vivo assays.

### Noncanonical miRNA Functions

**Figure 4:**
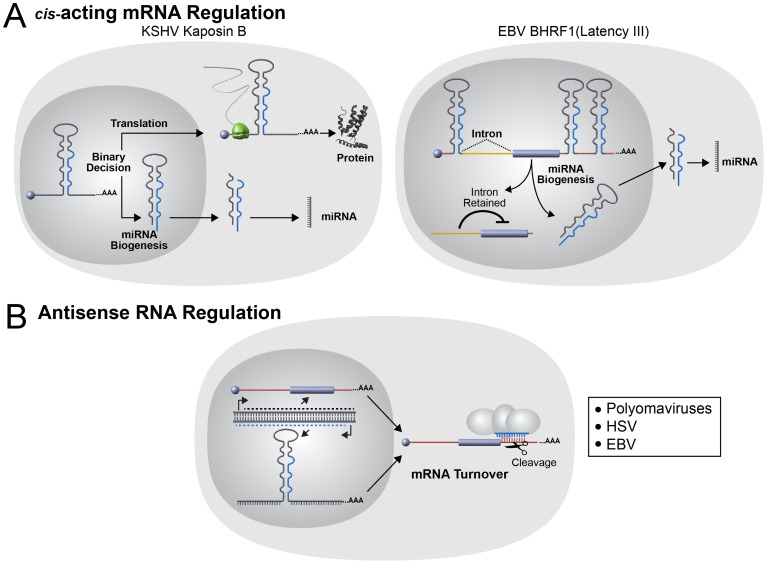
Noncanonical viral miRNA functions. (A) The use of host miRNA biogenesis machinery to modulate mRNA in *cis* has been reported in two different herpesviruses. On the left, the KSHV gene Kaposin B (KapB) functions as both an mRNA encoding a protein and a pri-miRNA for two KSHV-encoded miRNAs. Each KapB transcript may function as either an mRNA or pri-miRNA, but not both since pre-miRNA biogenesis occurs in the nucleus and destroys the mRNA. The levels of the host Microprocessor component Drosha is decreased during lytic replication and thus allows a shift to the pathway favoring KapB protein production [104]. On the right, the EBV gene BHRF1 functions as both an mRNA encoding a protein and a pri-miRNA for multiple EBV-encoded miRNAs. During Latency III, Microprocessor binds the transcript and the 5′ translation inhibitory intron is retained in BHRF1 transcripts. Altered transcription start site selection during the lytic cycle results in altered splicing and expression of the BHRF1 protein [105]. The net result is little BHRF1 protein expression occurs in latency with increased expression during lytic infection. (B) Antisense miRNA/mRNA interactions have been reported in both polyomaviruses and herpesviruses. Viral miRNAs are encoded antisense to viral mRNAs and the viral miRNAs direct siRNA-like cleavage of the mRNAs transcribed in the sense orientation. This antisense arrangement may not only decrease levels of the mRNA but may also serve as a posttranscriptional insulator in *cis* and *trans* to prevent the accumulation of long antisense RNAs.

Finally, for virus-encoded miRNAs, especially those that are not analogs of host miRNAs, all possible functions must be considered. Several viral miRNAs are found clustered in genomic regions near origins of replication or antisense to ncRNAs. It is possible that completely novel functions in genome replication or regulation of ncRNAs await discovery for viral and host miRNAs. In addition to canonical miRNA repressive activities on *trans* targets, the biogenesis of some miRNAs can convey *cis* regulation [103]. Several viruses encode pre-miRNAs embedded in *cis* within viral mRNA transcripts (Figure 4A). Pre-miRNAs from KSHV and EBV have been shown to function as negative *cis* regulators of viral protein expression albeit via different mechanisms. In KSHV two pre-miRNAs embedded within the Kaposin B (KapB) 3′UTR are cleaved by the Microprocessor complex resulting in decreased KapB protein expression in latency [104]. Interestingly, this repression is partially alleviated during lytic replication implicating a posttranscriptional mechanism for differential control of viral gene expression in latent versus lytic infection. The EBV BHRF1 transcript is also regulated by *cis* pre-miRNA elements, but in this situation, docking of the Microprocessor complex combined with an alternative transcription initiation site is hypothesized to promote an altered splicing pattern of this mRNA to a form that is subject to reduced translational efficiency [105]. Thus, although via different mechanisms, the net result is similar to KapB with less BHRF1 protein being produced specifically during latency. As viruses often serve as divining rods pointing towards new host activities, such seemingly atypical activities of viral miRNAs likely apply to some host transcripts as well.

## The Future

Currently, specific challenges and goals of the virus-encoded miRNA field overlap extensively with the broader parent fields of both virology and RNAi. Similar to host miRNAs, it will be imperative to determine which of the reported viral miRNAs possess biologically relevant activities. Several reported viral miRNAs are expressed at low levels compared to the other host and viral miRNAs. Are these miRNAs expressed at higher levels in other contexts, or do they possibly function at lower levels by a currently unknown mechanism? With notable exceptions, there is a striking lack of evolutionary conservation of most viral miRNAs. This could imply that viral miRNAs are a site of rapid evolution, perhaps even a driver of speciation. To help better understand which viral miRNAs are most relevant, it would be useful to have a deeper survey of the viruses that encode miRNAs. Perhaps even more informative will be to understand why some members of the same virus subfamilies do and do not encode miRNAs (e.g., HTLV in the Delta Retroviridae or VZV in the Varicello Herpesviridae [36], [46]). Of course, the overarching goal of the field is to parlay the survey of bona fide miRNAs into an understanding of their function.

In terms of understanding viral miRNA function, two classes emerge: those that mimic host miRNAs (analogs) and those that are viral specific (Figure 3). Mimicking host miRNAs provides obvious benefits to a virus by allowing it to access a pre-existing target network of numerous host transcripts that may have been selected for a particular functional outcome (e.g., prevention of apoptosis or evasion of immune signaling). In this regard, it seems curious that more examples of viral miRNA analogs of host miRNA do not exist as our estimates suggest that the majority of viral miRNAs do not share seed identity with bona fide host miRNAs (Figure 3A). Despite this, several recent high throughput target identification studies demonstrate that numerous host transcripts are directly bound by nonanalog viral miRNAs [62], [92], [106]. This raises several questions. How are these miRNAs targeting so many transcripts, and which of these interactions are biologically relevant? Certainly, some of these miRNAs may be tapping into existing miRNA target sites or other *trans* factor docking site networks by unknown mechanisms. For other nonanalog viral miRNAs, it seems unlikely that all identified targets are advantageous, especially given that introduction of some nonnatural siRNAs into cells will also redirect RISC to a similarly large number of unintended targets [107]. More likely, on an individual basis, some reported viral miRNA targets could be neutral or even provide a negative fitness cost to the virus as long as the sum total of negatively regulated targets remains of overall advantage to the virus. A challenge for the field will be extracting from these very valuable lists of viral miRNA-RISC-bound targets those that are the most functionally relevant.

Another mystery that applies to animal host and viral miRNAs is the observation that most lack perfect complementarity to their target transcripts. It has been hypothesized that miRNAs evolved independently in plants and animals [18]. In plants, for unknown reasons, most of the known miRNA targets are bound with perfect complementarity, resulting in siRNA-like RISC-mediated cleavage of the target transcripts. Some exceptional viral miRNAs do bind with perfect complementarity and direct cleavage of their targets, but this is uncommon and restricted to transcripts that lie antisense to the miRNA as opposed to cleaving host targets (Figure 4B). The reason why miRNA-mediated cleavage is not employed more often by viral or animal host miRNAs is unknown. Clearly, mammalian RISC can be programmed to direct siRNA-like cleavage of mRNA transcripts in a laboratory setting. It is possible that siRNAs work in mammalian cells because they access a vestigial remnant from when RNAi played a predominant antiviral role. Even so, this model would predict that at least some viral miRNAs would have evolved to utilize perfectly complementary miRNAs to more robustly eliminate undesirable host transcripts. On the other hand, it could be that Ago-mediated mRNA cleavage is somehow disadvantageous to the virus or host cells. For example, Ago-mediated cleavage could signal a damage response. In this regard, it is interesting to note that siRNAs perfectly complementary to some transposons have been cloned in mammalian cells and various components of the RNAi machinery are linked to sensing and suppressing transposon activity [11], [12], [108], [109].

Finally, a major reason for studying virus-encoded miRNAs is to be able to better develop therapeutic interventions. One of the most exciting stories in the world of viruses and miRNAs comes not from a virus-encoded miRNA but rather a host miRNA, miR-122 [25]. miR-122 is essential for maximal replication of HCV, and strategies to hinder HCV replication based on blocking this miRNA are already showing promise in both in vivo models and preliminary clinical studies [110]. These studies inspire hope that targeting virus-encoded miRNAs may also be clinically viable. As is true for all miRNA therapeutic applications, the main hurdle will be delivery of inhibitors or mimics to the appropriate tissues. If this hurdle can be surmounted, it is then plausible that blocking viral miRNAs could be used as a strategy to purge the latent reservoir—something of a “holy grail” in the herpesvirus field. Determining if such strategies are possible and uncovering which miRNAs are the most promising targets will require us to greatly advance our current understanding of viral miRNA functions. These could include canonical as well as noncanonical miRNA functions (for example, regulating origins of replication or modifying chromatin). Ultimately, a true understanding of viral miRNA function will require additional animal studies and likely the development of new relevant animal models for persistent infections. With the development of these new tools and models, viruses will continue to provide insights into host miRNA pathways and reveal new targets and functions of interest and possibly clinical relevance.

## Notes

While this manuscript was in revision, three additional papers documenting functions of KSHV miRNAs were published [111]–[113]. These papers further support the model that viral miRNAs contribute to cell longevity and modulation of the immune response. In addition, a paper documenting the Microprocessor complex in regulation of RNA pol II transcription through binding pri-miRNA-like sequences was also published [114]. This paper provides an additional example of a noncanonical *cis*-regulation mediated by components of the miRNA pathway.
